# Supplementary material for: An order-to-disorder structural switch activates the FoxM1 transcription factor
Source: eLife. 2019 May 28;8:e46131. doi: 10.7554/eLife.46131 (PMC6538375; doi:10.7554/eLife.46131)
Supplement: Supplementary file 1. [file elife-46131-supp1.docx]

|  |  | K_d_ (μM) | n | ΔH (kcal/mol) |  |
| --- | --- | --- | --- | --- | --- |
| FoxM1 1-203 | FoxM1 526-748 | 2 ± 1 | 0.96 ± 0.04 | -7 ± 2 | Fig. 1C |
| FoxM1 1-114 | FoxM1 696-748 | 3 ± 1 | 0.98 ± 0.03 | -9 ± 3 | Fig. 1C |
| FoxM1 1-16 | FoxM1 696-748 | no heat | -- | -- | Fig. 1C |
| FoxM1 80-114 | FoxM1 696-748 | no heat | -- | -- | Fig. 1C |
| FoxM1 1-203 | FoxM1 694-726 | > 10 | -- | -- | Fig. 1C |
| FoxM1 1-203 | FoxM1 727-748 | no heat | -- | -- | Fig. 1C |
| FoxM1 1-117/694-748 | FoxM1 696-748 | no heat | -- | -- | Fig. 1C |
| FoxM1 1-203 | FoxM1 696-748 | 2.2 ± 0.4 | 0.98 ± 0.03 | -7 ± 3 | Fig. 3B |
| FoxM1 1-203 I62A/I64A | FoxM1 696-748 | 8.1 ± 0.8 | 1.01 ± 0.05 | -8.1 ± 0.2 | Fig. 3B |
| FoxM1 1-203 V76A/I78A | FoxM1 696-748 | no heat | -- | -- | Fig. 3B |
| FoxM1 1-203 F106A/L108A | FoxM1 696-748 | no heat | -- | -- | Fig. 3B |
| FoxM1 1-203 I65A | FoxM1 696-748 | 8 ± 2 | 1.04 ± 0.01 | -8.1 ± 0.1 | Fig. 3B |
| FoxM1 1-203 V75A | FoxM1 696-748 | 9 ± 2 | 0.98 ± 0.02 | -7 ± 3 | Fig. 3B |
| FoxM1 1-203 I107A/I109A | FoxM1 696-748 | 1.5 ± 0.4 | 1.00 ± 0.01 | -6.4 ± 0.1 | Fig. 3B |
| FoxM1 1-203 I88A | FoxM1 696-748 | no heat | -- | -- | Fig. 3B |
| FoxM1 1-203 L91A | FoxM1 696-748 | no heat | -- | -- | Fig. 3B |
| FoxM1 1-203 | FoxM1 696-748 V708A | no heat | -- | -- | Fig. 3B |
| FoxM1 1-203 | FoxM1 696-748 L709A | no heat | -- | -- | Fig. 3B |
| FoxM1 1-203 | FoxM1 696-748 M712A | 7.7 ± 0.1 | 1.02 ± 0.04 | -2.6 ± 0.1 | Fig. 3B |
| FoxM1 1-203 | FoxM1 696-748 L716A | no heat | -- | -- | Fig. 3B |
| FoxM1 1-203 + Cdk | FoxM1 526-748 | 6 ± 3 | 0.99 ± 0.04 | -5.9 ± 0.7 | Fig. 4A |
| FoxM1 1-203 | FoxM1 526-748 + Cdk | 2.1 ± 0.5 | 0.99 ± 0.01 | -2.3 ± 0.1 | Fig. 4A |
| FoxM1 1-114/526-674 | FoxM1 696-748 | 1.1 ± 0.1 | 0.7 ± 0.1 | -13.3 ± 0.1 | Fig. 4A |
| FoxM1 1-114/526-674 + Cdk | FoxM1 696-748 | 2.4 ± 0.9 | 0.98 ± 0.07 | -10 ± 3 | Fig. 4A |
| FoxM1 1-203 | FoxM1 696-748 + Plk1 | no heat | -- | -- | Fig. 4B |
| FoxM1 1-203 | FoxM1 696-748 3A^1^ | 4.0 ± 0.8 | 1.0 ± 0.1 | -4.2 ± 0.8 | Fig. 4B |
| FoxM1 1-203 | FoxM1 696-748 3A + Plk1 | no heat | -- | -- | Fig. 4B |
| FoxM1 1-203 | FoxM1 696-748 S715A | 5 ± 2 | 1.2 ± 0.5 | -8 ± 1 | Fig. 4B |
| FoxM1 1-203 | FoxM1 696-748 S715A +Plk1 | 4 ± 1 | 1.2 ± 0.3 | -8 ± 1 | Fig. 4B |
| CBP KIX | FoxM1 696-748 | 2.7 ± 0.5 | 0.9 ± 0.2 | -3.3 ± 0.5 | Fig. 6A |
| CBP TAZ2 | FoxM1 696-748 | 0.7 ± 0.3 | 0.95 ± 0.02 | -2.6 ± 0.2 | Fig. 6A |
| CBP TAZ1 | FoxM1 696-748 | no heat | -- | -- | Fig. 6A |
| CBP TAZ2 | FoxM1 1-117/694-748 | no heat | -- | -- | Fig. 6B |
| CBP TAZ2 | FoxM1 1-117/694-748 I88A | 6 ± 3 | 0.90 ± 0.04 | -1.8 ± 0.5 | Fig. 6B |
| CBP TAZ2 | FoxM1 526-748 | 2.0 ± 0.1 | 0.94 ± 0.06 | 2.7 ± 0.2 | Fig. 6C |
| CBP TAZ2 | FoxM1 526-748 + Cdk | 1.7 ± 0.2 | 0.86 ± 0.05 | 5.0 ± 0.7 | Fig. 6C |
| CBP TAZ2 | FoxM1 526-748 + Plk1 | 2.3 ± 0.3 | 0.72 ± 0.01 | 6.6 ± 0.6 | Fig. 6C |
| CBP TAZ2 | FoxM1 699-726 | no heat | -- | -- | Fig. 6D |
| CBP TAZ2 | FoxM1 727-748 | no heat | -- | -- | Fig. 6D |
| CBP TAZ2 | FoxM1 696-748 L716A | no heat | -- | -- | Fig. 6D |
| FoxM1 573-635 | Plk1 polobox | no heat | -- | -- | Fig. 4S2 |
| FoxM1 573-635 + Cdk | Plk1 polobox | 0.08 ± 0.07 | 0.88 ± 0.05 | -29 ± 3 | Fig. 4S2 |

**Supplementary File 1: Summary of ITC data fitting**

^1^ 3A is a S702A/S724A/S741A mutation
