# Supplementary material for: An order-to-disorder structural switch activates the FoxM1 transcription factor
Source: eLife. 2019 May 28;8:e46131. doi: 10.7554/eLife.46131 (PMC6538375; doi:10.7554/eLife.46131)
Supplement: Supplementary file 2. [file elife-46131-supp2.docx]

**Supplementary File 2: NMR restraints and structural statistics for NRD-TAD structural ensemble.**

| **Restraints** |  |
| --- | --- |
| Total NOE distance restraints | 64 |
| Intraresidue | 0 |
| Inter-residue | 64 |
| Sequential (\|*i* – *j*\| = 1) | 0 |
| Medium-range (\|*i* – *j*\| <= 4) | 2 |
| Long-range (\|*i* – *j*\| >= 5) | 62 |
| Interdomain | 17 |
| Hydrogen bonds | 0 |
| Total dihedral angle restraints ^a^ | 98 |
| φ | 49 |
| ψ | 49 |
| Total RDC restraints | 45 |
| **Structure statistics** |  |
| RDC Q_work_ (mean ± s.d.)^b^ | 0.37 ± 0.02 |
| RDC Q_free_ (mean ± s.d.)^b^ | 0.38 ± 0.04 |
| Violations (mean ± s.d.) |  |
| Distance constraints (Å)  between 1 Å and 2 Å/structure  between 2 Å and 3 Å/structure  between 3 Å and 4 Å/structure  above 4.0 Å/structure | 1.4 ± 1.1  1.1 ± 0.7  0.1 ± 0.3  0.0 ± 0.0 |
| Dihedral-angle violation (°)^c^  Max. dihedral angle violation (°) | 10.6 ± 1.3  34.11 |
| Deviations from idealized geometry  Bond lengths (Å)  Bond angles (°)  Impropers (°) | 0.0 ± 0.0  0.0 ± 0.0  0.0 ± 0.0 |
| Average pairwise r.m.s. deviation (Å) ^d^  Heavy  Backbone | 0.48  0.41 |

^a^ Residues that have good TALOS-N prediction. These residues exclude the TEV linker site.

^b^ Q_work_ is calculated from NH RDCs, which were used in the structure calculations. Q_free_ is calculated using NCO RDCs, which were not used in the structure calculations. See Figure 2-supplement figure 2.

^c^ Dihedral angle restraints were derived from TALOS-N. The violations were calculated for the core secondary structural regions of the final ensemble consisting of ten models using 15° cut-off beyond TALOS-N predicted dihedral angles.

^d^ R.M.S. deviation was calculated over the structured region consisting of α-helices and β-sheets in the final ensemble.
